# Identification of *Rf9*, a Gene Contributing to the Genetic Complexity of Fertility Restoration in Hybrid Wheat

**DOI:** 10.3389/fpls.2020.577475

**Published:** 2020-12-10

**Authors:** Fahimeh Shahinnia, Manuel Geyer, Annette Block, Volker Mohler, Lorenz Hartl

**Affiliations:** Bavarian State Research Centre for Agriculture, Institute for Crop Science and Plant Breeding, Freising, Germany

**Keywords:** cytoplasmic male sterility, seed set, sterile spikelet, *Triticum aestivum*, *Triticum timopheevii*

## Abstract

Wheat (*Triticum aestivum* L.) is a self-pollinating crop whose hybrids offer the potential to provide a major boost in yield. Male sterility induced by the cytoplasm of *Triticum timopheevii* is a powerful method for hybrid seed production. Hybrids produced by this method are often partially sterile, and full fertility restoration is crucial for wheat production using hybrid cultivars. To identify the genetic loci controlling fertility restoration in wheat, we produced two cytoplasmic male-sterile (CMS) backcross (BC_1_) mapping populations. The restorer lines Gerek 79 and 71R1203 were used to pollinate the male-sterile winter wheat line CMS-Sperber. Seed set and numbers of sterile spikelets per spike were evaluated in 340 and 206 individuals of the populations derived from Gerek 79 and 71R1203, respectively. Genetic maps were constructed using 930 and 994 single nucleotide polymorphism (SNP) markers, spanning 2,160 and 2,328 cM over 21 linkage groups in the two populations, respectively. Twelve quantitative trait loci (QTL) controlled fertility restoration in both BC_1_ populations, including a novel *restorer-of-fertility* (*Rf*) locus flanked by the SNP markers *IWB72413* and *IWB1550* on chromosome 6AS. The locus was mapped as a qualitative trait in the BC_1_ Gerek 79 population and was designated *Rf9*. One hundred-nineteen putative candidate genes were predicted within the QTL region on chromosome 6AS. Among them were genes encoding mitochondrial transcription termination factor and pentatricopeptide repeat-containing proteins that are known to be associated with fertility restoration. This finding is a promising step to better understand the functions of genes for improving fertility restoration in hybrid wheat.

## Introduction

Since the discovery of male sterility and restoration systems in the 1960s, hybrid wheat triggered attention due to its potential for improved grain and straw productivity and yield stability particularly under harsh and marginal environments (Longin et al., 2012). The major gains of hybrid vs. line varieties are improved trait values due to heterosis (Castillo et al., 2014). Hybrid wheat has been reported to provide uniform plant establishment and tolerance against frost, lodging, and diseases such as leaf rust, stripe rust, *Septoria tritici* blotch, and powdery mildew (Gupta et al., 2019).

To harness yield gains associated with hybrid vigor, the cytoplasmic male sterility (CMS) system provides a cost-effective tool for efficient hybrid seed production (Chen and Liu, 2014). CMS in plants is based on the rearrangements of mitochondrial DNA that lead to chimaeric genes and a condition under which a plant is unable to produce fertile pollen (Eckardt, 2006; Whitford et al., 2013). CMS evades the need for manual removal of anthers, thus facilitating a technology to produce unlimited numbers of hybrid plants. It has been successfully used in crops such as rye, rice, maize, and sunflower (Castillo et al., 2015). The use of *T. timopheevii* cytoplasm in bread and durum wheat creates male sterility, whereas female fertility is not impaired. Wilson and Ross (1962) were the first to describe a workable cytoplasmic male sterile of *T. timopheevii* with largely neutral effects on the agronomic and quality characteristics. This CMS system has gained widespread use due to the deleterious effects of other cytoplasms of the genera *Triticum* and *Aegilops* on various traits, and because no advantage existed over the *T. timopheevii* system (Virmani and Edwards, 1983). It uses three different breeding lines: a CMS line, maintainer line, and restorer line. The CMS line is used as the female parent with at least one CMS-causing gene in the *T. timopheevii*-derived cytoplasm and lacking functional nuclear-encoded *Restorer-of*-*fertility* (*Rf*) genes (Schnable and Wise, 1998). The maintainer line serves as the male parent in crosses for the propagation and maintenance of the CMS line, with the same nuclear genome as the CMS line but a normal fertile *T. aestivum* cytoplasm. The restorer line retains (a) functional *Rf* genes and acts as the male parent to cross with the CMS line to produce the F_1_ hybrid seeds. In F_1_ plants, the *Rf* genes restore male fertility, and the combination of the nuclear genomes from the CMS line and the restorer line produces hybrid vigor. For commercial hybrid seed production, a male-sterile line has to be crossed with a line carrying dominant restorer alleles and suitable pollinator qualities (Whitford et al., 2013). While fertility restoration is a crucial trait in hybrid breeding, hybrids produced using this method are often partially sterile due to the complex interaction between the mitochondrial and nuclear genes controlling male specificity and restoration of fertility (Chen and Liu, 2014). Therefore, incomplete fertility restoration poses a major bottleneck for hybrid wheat breeding, as it compromises the heterotic gain for grain yield and the uniformity or quality of end-use products.

Fertility restoration is a genetically complex process and is mainly controlled by the mitochondrial genome in interaction with *Rf* genes (Eckardt, 2006). Besides, it is known that fertility restoration is influenced by environmental factors including photoperiod, water stress, light intensity, and temperature (Johnson et al., 1967). The nuclear encoded gene families that act in the mitochondria produce proteins that share the common structural organization of similar repeated helical motifs and include pentatricopeptide repeat (PPR) proteins and mitochondrial transcription termination factors (mTERFs) (Pan et al., 2019). The effect of cytoplasmic male sterility can be suppressed by preventing the accumulation of mitochondrial encoded CMS-conferring gene products through the function of a class of *Rf* genes, generally belonging to a large family of genes that encode organelle-targeted PPR proteins (Hu et al., 2012). mTERF genes are widely distributed in metazoans, plants, and green alga. They regulate transcription, translation, and DNA replication of mitochondrial genes in metazoans while regulating gene expression in chloroplasts and mitochondria in plants (Pan et al., 2019). In wheat, the presence of eight major loci (*Rf1-Rf8*) for *timopheevii*-based cytoplasmic male sterility is known and assigned to the chromosomes 1A, 7D, 1B, 6B, 6D, 5D, 7B, and 2D, respectively (Mukai and Tsunewaki, 1979; Sinha et al., 2013; Gupta et al., 2019). *Rf1* and *Rf3* are the most effective genes for achieving restoration in wheat (Geyer et al., 2016, 2018; Würschum et al., 2017). Previous studies have indicated that combinations of two or three major *Rf* genes and restorer genes with small effect or low penetrance (modifier loci) can modify the degree of fertility restoration (Ma et al., 1995; Ahmed et al., 2001; Zhou et al., 2005; Stojałowski et al., 2013). Consequently, attempts are made to pyramid multiple dominant or partially dominant alleles of the most favorable genes or quantitative trait loci (QTL), including those involved in epistatic interactions to achieve complete fertility restoration in hybrid wheat (Gupta et al., 2019). Understanding the genetic mechanisms underlying restoration of fertility and developing elite restorer lines are crucial to overcome the intricate barriers in hybrid breeding programs. For this reason, our objective was to identify new genetic loci controlling fertility restoration that can be employed in hybrid wheat breeding. Here, we developed two CMS-based backcross (BC) mapping populations, which we used for QTL mapping and identification of candidate genes. Our study identified a new *Rf* locus (*Rf9*) and novel QTL for seed set and number of sterile spikelets on chromosomes 1DS, 2AL, 4AL, 5BL, and 6AS. Putative candidate genes located in the target regions are discussed.

## Materials and Methods

### Plant Materials and Population Development

Two BC_1_ mapping populations were developed using Gerek 79 and 71R1203 as fertility-restoring parental lines. The winter wheat cultivar Gerek 79 (PI 559560, pedigree: Mentana/Mayo-48//4-11/3/Yayla-305) originated in 1979 from the Transitional Zone Agricultural Research Institute, Anadolu ARI, Eskisehir, Turkey^1^. The restoration capacity of Gerek 79 was found in initial screening experiments (unpublished) when pollinating CMS-Sperber with cultivars from various regions and testing the hybrids for self-fertility in the greenhouse at the Bavarian State Research Center for Agriculture (LfL). The winter wheat restorer line 71R1203 (PI 473552, pedigree: NB542437/CI 13438//2^∗^Burt/3/NB542437/2^∗^CI13438) was developed in 1982 by the USDA-ARS and Washington State University. The specific fertility restoration loci possessed by each of the two sources have not been previously determined, but 71R1203 was known to potentially carry restorer loci *Rf1* and *Rf2* that are present in NB542437 (Allan and Rubenthaler, 1984). The variety Sperber (registered 1983) and CMS-Sperber are maintained at the LfL. Seeds of Gerek 79 were kindly provided by Prof. Friedrich Zeller (Technical University of Munich, Freising, Germany) and are available at the Germplasm Resources Information Network (GRIN), U.S. National Plant Germplasm System. Seeds of the line 71R1203 were obtained from the National Small Grains Collection, US. Gerek 79 and 71R1203 were used as restorer lines in crosses with the male-sterile winter wheat line CMS-Sperber. The hybrids were then backcrossed with the maintainer line Sperber to develop the mapping populations CMS-Sperber/Gerek 79//Sperber (BC_1_ Gerek 79) and CMS-Sperber/71R1203//Sperber (BC_1_ 71R1203).

### Field Trials and Phenotyping

The BC_1_ Gerek 79 population was vernalized in a climate chamber at 6°C for 8 weeks and planted in spring 2019 in an LfL field at Freising (48°24′12.64″N, 11°44′55.54″E), Germany. The BC_1_ 71R1203 population was sown in autumn 2018 in the field at KWS LOCHOW GMBH in Bergen (52°48′30.13″N, 9°57′49.46″E), Germany. We used non-replicated trials for assessing the fertility restoration due to having mortal mapping populations. Four emerging spikes from the main tillers of each BC_1_ line were covered before anthesis using glassine bags. After ripening, the spikes were harvested and the seed set (as the restored fertility trait) and number of sterile spikelets per spike (as the non-restored fertility trait) were counted. The seed set of a plant was calculated as the number of kernels divided by the number of spikelets, averaged over all four bagged spikes per individual. Plants were considered fertile if they had at least one seed per spike and male sterile when no seed was produced. Observed ratios of fertile to sterile plants in each mapping population were tested against the expected segregation pattern using the *chi*-*squared goodness-*of-*fit test*. Statistical analyses including descriptive statistics, correlation, and frequency distribution of the traits were conducted in the SigmaPlot (Systat Software, San Jose, CA, United States).

### Genotyping and Linkage Analysis

Genomic DNA of parental lines and BC_1_ progenies was extracted from young leaf tissues following the procedure of Plaschke et al. (1995). Based on the fertility restoration data, the DNA of 273 and 184 individuals from BC_1_ Gerek 79 and BC_1_ 71R1203, respectively, were selected for genotyping using a bead chip comprising 16,762 single nucleotide polymorphism (SNP) markers selected from the 90K iSelect^®^ array (Wang et al., 2014). SNP genotyping was done by KWS SAAT S & Co., KGaA, Einbeck, Germany. The raw SNP data were analyzed as described by Geyer et al. (2018). Briefly, all monomorphic SNPs and those with more than 10% missing values and a minor allele frequency of less than 10% were discarded from further analysis using the synbreed package V0.12-6 (Wimmer et al., 2012) in R (R Core Team, 2017). Linkage analysis was done using JoinMap^®^ (Kyazma BV, Wageningen, Netherlands). The Kosambi mapping function (Kosambi, 1944) was used to convert the recombination frequencies into centimorgans (cM).

To determine whether Gerek 79 and 71R1203 carried *Rf3*, they were genotyped with SNP marker *IWB72107*, earlier shown to have a high potential for predicting *Rf3* (Geyer et al., 2016). The SNP-containing sequence for *IWB72107* was retrieved from The Triticeae Toolbox (T3^2^) and converted to a Kompetitive Allele Specific Polymerase (KASP) chain reaction marker assay (Supplementary Table S1). Plants were genotyped according to the manufacturer’s instructions (LGC Genomics, Hoddeson, United Kingdom). Each KASP reaction was prepared in a volume of 10 μL with 5 μL DNA and 5 μL of the genotyping master mix. Amplification was carried out using the CFX96 Touch Real-Time PCR SNP Detection System (Bio-Rad, Hercules, CA, United States), starting with 15 min at 94°C, followed by 40 cycles of PCR with 94°C for 20 s and 65°C for 1 min and 10 cycles of touch down PCR where the annealing temperature was gradually reduced by 0.8°C per cycle. Endpoint analysis and allelic discrimination related to SNP calls were accomplished using the CFX96 Touch^TM^ software (Bio-Rad, Hercules, CA, United States). The DNA of the restorer line Primepi was used as a reference control for *Rf3* (Geyer et al., 2016).

### QTL Mapping

To detect the QTL controlling seed set and number of sterile spikelets per spike in BC_1_ populations, composite interval mapping with a 5-cM window and a maximum of 10 marker cofactors per model was carried out using the Windows QTL Cartographer version 2.5 (Wang et al., 2012). Tests were performed at 1-cM intervals, and cofactors were selected by the forward-backward stepwise regression Model 6 (Shahinnia et al., 2009; Wang et al., 2012). Genome-wide, trait-specific threshold values (α = 0.05) of the likelihood ratio test statistic for declaring the presence of a significant QTL was determined by 2,000 permutations (Churchill and Doerge, 1994). The additive effect of an allelic substitution at each QTL and the phenotypic variation explained by a QTL (*R*^2^) conditioned by the composite interval mapping cofactors involved in the model was calculated at the most likely QTL position. The LOD peak of each significant QTL was reflected as the QTL location on the linkage map. To identify markers associated with trait variation located in the confidence interval of a target QTL, single marker analysis was performed using Wald statistics (Kenward and Roger, 1997; Shahinnia et al., 2016). QTL designation followed the recommended rules for wheat^3^. QTL nomenclature (*Qphenotype.lab-chromosome.Qnumber*) included “lfl,” “Rf,” and “StS” representing ‘Bayerische Landesanstalt für Landwirtschaft’ (LfL), seed set, and number of sterile spikelets per spike, respectively. The *Qnumber* after the chromosome designation refers to overlapping QTL identified on the same chromosome in the two BC_1_ populations.

### Physical Mapping and Identification of Candidate Genes

The sequences of the *QRf.lfl-6AS.1* flanking markers *IWB72428* (3.8 cM) and *IWB841* (6.5 cM) within the detected region, that were up to 2 LOD drops from the maximum likelihood value of the selected QTL, were aligned to the reference sequence of Chinese Spring (IWGSC RefSeq v.1.0, Appels et al., 2018) by BLASTN through the URGI portal^4^ to identify the physical position of the QTL. The protein sequences of the genes in the QTL interval were obtained from Ensembl Plants^5^ and used for BLASTP homology search (Adamski et al., 2020). Descriptions for the wheat predicted genes based on the IWGSC RefSeq v.1.1 were obtained from BioMart^6^.

*In silico* expression values for tissue series of the wheat spike, root, leaf, grain, and stem organs at different developmental stages (Zadoks et al., 1974) were obtained through the WheatExp^7^ (Pearce et al., 2015) and a bread wheat tissue series RNA-Seq data set^8^ in POTAGE (Suchecki et al., 2017). Fragments per kb per million reads (FPKM) were used to show the gene expression quantity, thus avoiding the influence of sequencing length and differences on expression values.

## Results

### Evaluation of Fertility Restoration

**FIGURE 1:**
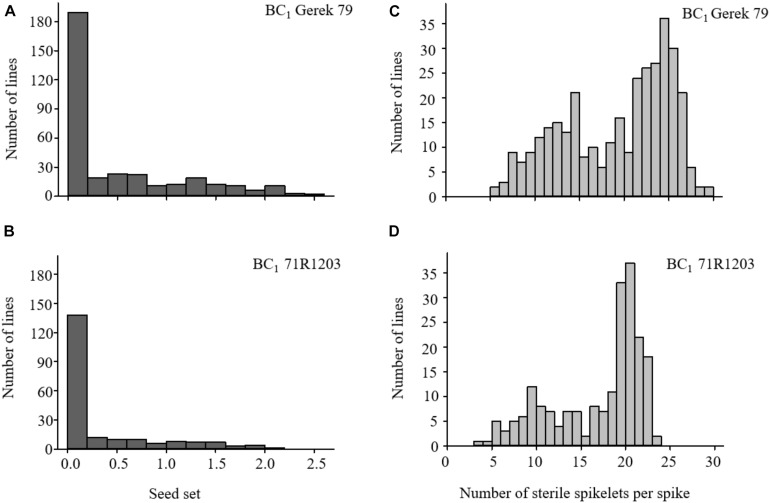
Frequency distribution of phenotypes for **(A,B)** seed set and **(C,D)** number of sterile spikelets per spike in BC_1_ Gerek 79 (*n* = 340 lines) and BC_1_ 71R1203 (*n* = 206 lines), respectively. Data are shown based on average overall four spikes (*n* = 4) per BC_1_ line.

The values for seed set and number of sterile spikelets per spike in BC_1_ Gerek 79 and BC_1_ 71R1203 are presented in Table 1. Whereas a 1:1 segregation ratio for fertile to sterile lines was observed in both BC_1_ Gerek 79 (174:166) and BC_1_ 71R1203 (100:106), the average seed set was higher in BC_1_Gerek 79 (0.5) than in BC_1_ 71R1203 (0.3). Number of sterile spikelets per spike showed a negative correlation with seed set in both BC_1_ Gerek 79 (*r* = −0.65) and BC_1_ 71R1203 (*r* = −0.87). Frequency distribution of the traits ranged between 0–2.5 and 0–2.1 for seed set (Figures 1A,B), whereas a range between 5.5–29.5 and 3.7–23.0 was observed for number of sterile spikelets per spike (Figures 1C,D) in BC_1_ Gerek 79 and BC_1_ 71R1203, respectively.

**TABLE 1:** Descriptive statistics for seed set and number of sterile spikelets per spike in BC_1_ Gerek 79 and BC_1_ 71R1203.

Trait/Population	No. lines	Mean	Max	Min	SE	No. fertile:sterile	χ*^2^-*test (*p* < 0.01)
BC_1_ Gerek 79	340					174:166	0.76^*ns*^
Seed set		0.5	2.5	0	0.03		
Number of sterile spikelets per spike		19.1	29.5	5.5	0.32		
BC_1_ 71R1203	206					100:106	0.77^*ns*^
Seed set		0.3	2.1	0	0.02		
Number of sterile spikelets per spike		16.8	23	3.7	0.31		

*Segregation of the number of fertile vs. sterile plants was compared to a 1:1 ratio using the χ*^2^-*test. ns, not significant.*

### Construction of Genetic Maps

Following filtration of 16,762 SNPs used for genotyping of BC_1_ lines, the resulting genetic base maps consisted of 929 and 994 unique SNP loci, spanning 2,160 and 2,328 cM over 21 linkage groups in BC_1_ Gerek 79 and BC_1_ 71R1203, respectively. The average distance (2.4 cM) between two unique loci was similar in both linkage maps (Supplementary Tables S2, S3).

**FIGURE 2:**
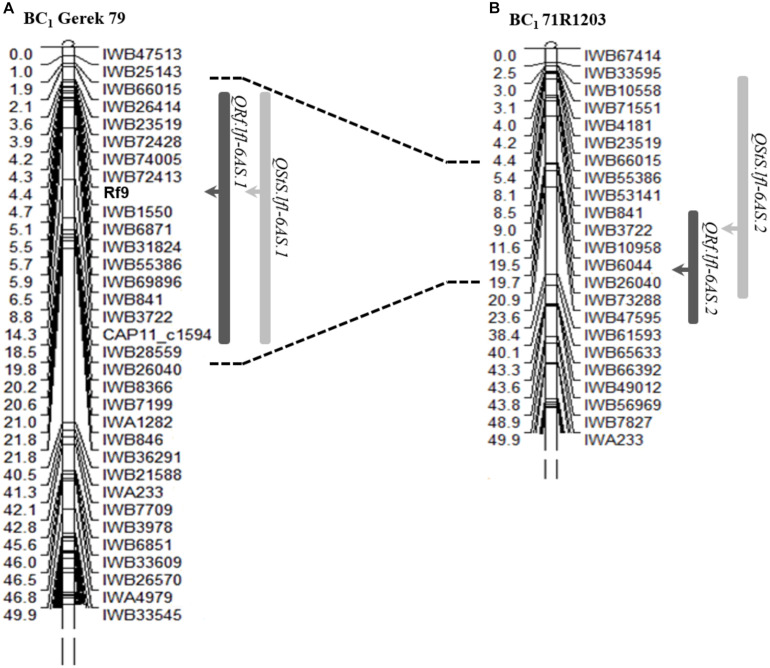
Genetic position of the gene *Rf9* and co-location of QTL detected for seed set (*QRf*, dark bars) and number of sterile spikelets per spike (*QStS*, light bars) on chromosome 6AS in **(A)** BC_1_ Gerek 79 and **(B)** BC_1_ 71R1203. The peak of each QTL is shown with arrows. Correspondence interval of *IWB66015-IWB26040* common markers in two maps is shown with dot lines. A partial linkage map of the short arm of chromosome 6A is presented.

Using the categorical fertility phenotypes (completely sterile or fertile), a new restorer locus was mapped as a qualitative (monogenically inherited) trait between SNP markers *IWB72413* (4.3 cM) and *IWB1550* (4.7 cM) in the subtelomeric region of chromosome 6AS in BC_1_ Gerek 79 (Figure 2A). The newly dissected locus was designated *Rf9* following the Catalog of Gene Symbols for Restorers for Cytoplasmic Male Sterility in wheat^9^. No restorer locus underlying the binary phenotype could be genetically mapped in BC_1_ 71R1203.

### Identification of QTL in BC_1_ Gerek 79 and BC_1_ 71R1203

Composite interval mapping in BC_1_ Gerek 79 (Table 2) detected two QTL for seed set on chromosomes 6AS (*QRf.lfl-6AS.1*) and 4AL (*QRf.lfl-4AL*) that explained 18 and 14% of the phenotypic variation, respectively. At both loci, the parental line Sperber contributed with negative additive effects indicating that the Gerek 79 alleles increased seed set values. In this population, three QTL for number of sterile spikelets per spike were identified on chromosomes 6AS (*QStS.lfl-6AS.1*), 6BS (*QStS.lfl-6BS*), and 2AL (*QStS.lfl-2AL*). Of these, *QStS.lfl-6AS.1*, located close to *IWB72428*, showed the highest LOD score (46.3) and explained 53% of the total phenotypic variation for number of sterile spikelets per spike. The QTL allele that increased the number of sterile spikelets was inherited from the parental line Sperber (Table 2).

**TABLE 2:** Chromosomal locations, map intervals, flanking markers, LOD scores, additive effects (A.E.) of Sperber, and percentage of explained variance by QTL detected for seed set (*QRf*) and number of sterile spikelets per spike (*QStS*) in BC_1_ Gerek 79 and BC_1_ 71R1203.

Trait/Population	QTL	Chromosome	Interval (cM)	Flanking markers	LOD	*R*^2^ (%)	A.E.
BC_1_ Gerek 79							
Seed set	*QRf.lfl-4AL*	4AL	48.6–50.7	*BS00062059-IWB74057*	3.1	14	–0.46
	*QRf.lfl-6AS.1*	6AS	1.8–14.2	*IWB66015-CAP11_c1594*	6.2	18	–0.34
Number of sterile spikelets per spike	*QStS.lfl-2AL*	2AL	46.7–47.3	*IWB12381-IWB53795*	3.1	9	2.07
	*QStS.lfl-6AS.1*	6AS	1.8–14.2	*IWB66015-CAP11_c1594*	46.3	53	8.59
	*QStS.lfl-6BS*	6BS	0–34	*IWA921-IWB12660*	14	21	6.35
BC_1_ 71R1203							
Seed set	*QRf.lfl-1AS*	1AS	1.6–20.7	*IWB7436-IWB28549*	7.2	12	–0.26
	*QRf.lfl-1BS*	1BS	0–10.2	*IWB69597-IWB12475*	4.4	7	–0.21
	*QRf.lfl-5BL*	5BL	133.1–147.1	*IWB33310-IWB26869*	3.6	5	–0.19
	*QRf.lfl-6AS.2*	6AS	9.0–23.5	*IWB3722-IWB47595*	5.9	11	–0.21
Number of sterile spikelets per spike	*QStS.lfl-1AS*	1AS	1.6–22.9	*IWB7436-IWB31602*	26.3	46	6.11
	*QStS.lfl-1DS*	1DS	2.5–21.8	*IWB11524-IWB59019*	3.6	4	2.29
	*QStS.lfl-6AS.2*	6AS	2.5–20.8	*IWB33595-IWB73288*	5.8	12	2.73

Four QTL for seed set in BC_1_ 71R1203 population (Table 2) were identified on chromosomes 1AS (*QRf.lfl-1AS*), 1BS (*QRf.lfl-1BS*), 5BL (*QRf.lfl-5BL*), and 6AS (*QRf.lfl-6AS.2*), explaining 12, 7, 5, and 11% of the total phenotypic variation, respectively. A higher seed set was conferred by the 71R1203 allele at all loci. The most significant QTL for number of sterile spikelets per spike with a LOD score of 26.3 was detected on chromosome 1AS (*QStS.lfl-1AS*) near to *IWB7436* with a positive additive effect derived from Sperber. This QTL, together with two QTL on chromosomes 1DS (*QStS.lfl-1DS*) and 6AS (*QStS.lfl-6AS.2*), explained 46, 4, and 12%, respectively, of the total phenotypic variation for the trait (Table 2).

**FIGURE 3:**
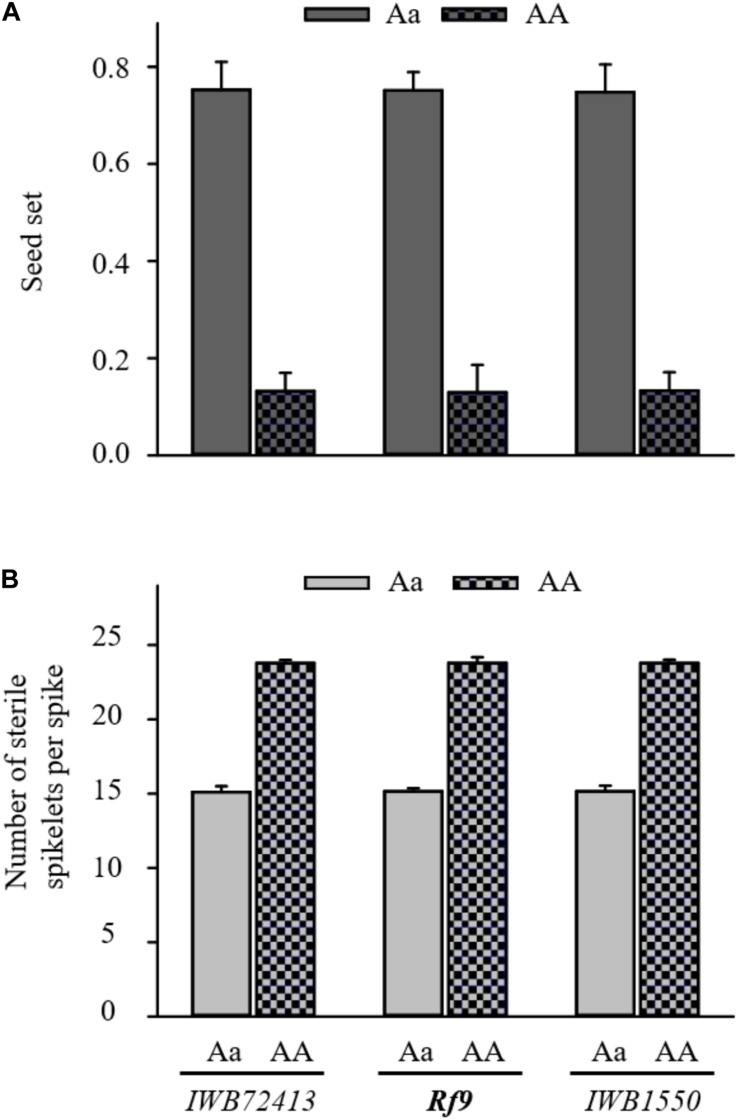
Comparison of allele effects for *Rf9* locus and two flanking SNP loci (*IWB72413* and *IWB1550*) at the peak of *QRf.lfl-6AS.1* and *QStS.lfl-6AS.1* on **(A)** seed set and **(B)** number of sterile spikelets per spike in BC_1_ Gerek 79. Dotted bars refer to the alleles of recurrent parent Sperber (AA) *vs*. heterozygous BC_1_F_1_ lines (Aa) in grey. T-tests based on the mean value of each trait showed highly significant differences (*p* < 0.001) between the allele effects of Sperber compared to Gerek 79 for all loci.

The QTL hotspot for seed set and number of sterile spikelets per spike on chromosome 6AS found in both populations was mapped to the same genomic region (Figures 2A,B). In BC_1_ Gerek 79, the QTL were identified in the interval between *IWB66015* (1.8 cM) and *CAP11_c1594* (14.2 cM) for controlling seed set (*QRf.lfl-6AS.1*) and number of sterile spikelets per spike (*QStS.lfl-6AS.1*) with the opposite allelic effect of Sperber (−0.34 and 8.59, respectively) (Table 2). Remarkably, the peak of both QTL harbored *Rf9*, located 4.4 cM proximal to the subtelomeric region in BC_1_ Gerek 79 (Figure 2A). The magnitudes and directions of allelic effects at *Rf9* and SNP loci *IWB72413* (4.3 cM) and *IWB1550* (4.7 cM) showed a highly significant effect for seed set (Figure 3A) and number of sterile spikelets per spike (Figure 3B), with the favorable allele derived from Gerek 79. In BC_1_ 71R1203, the QTL for seed set and number of sterile spikelets per spike (Figure 2B) revealed negative (−0.21 for *QRf.lfl-6AS.2*) and positive (2.73 for *QStS.lfl-6AS.2*) allelic effects of Sperber for seed set and number of sterile spikelets per spike, respectively.

### Candidate Genes Associated With *QRf.lfl-6AS.1*

**FIGURE 4:**
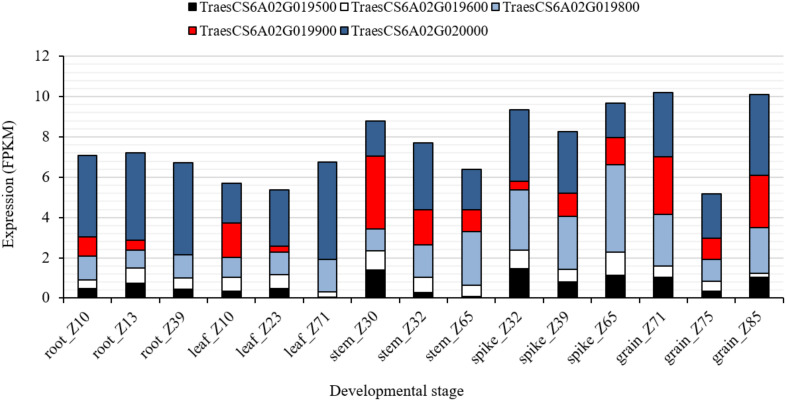
*In silico* expression analysis of the candidate genes *TraesCS6A02G019500*, *TraesCS6A02G019600*, *TraesCS6A02G019800, TraesCS6A02G019900*, and *TraesCS6A02G020000* encoded for mTERF in different wheat plant organs at different developmental stages according to Zadoks et al. (1974).

To further investigate the chromosomal region associated with *QRf.lfl-6AS.1*, harboring *Rf9*, SNP markers *IWB72428* (6A: 6.59 Mbp) and *IWB841* (6A: 12.39 Mbp) were used to search for putative candidate genes (Supplementary Table S4). The search resulted in 119 gene sequences physically located in nearly 5.8 Mbp on chromosome 6A (6.59–12.38 Mbp). Among those, 23 and 9 genes belonged to the mTERF and PPR family, respectively. The genes *TraesCS6A02G019500*, *TraesCS6A02G019600*, *TraesCS6A02G019800, TraesCS6A02G019900*, and *TraesCS6A02G020000* encoded for mTERF family physically (9.3 Mbp) located in the peak of *QRf.lfl-6AS.1* close to *IWB1550*, the flanking marker of *Rf9*. The *in silico* expression analysis of these genes showed a wide range of expression in different organs and at three developmental stages (Figure 4). The gene *TraesCS6A02G019800* was highly expressed (4.4 FPKM) at Zadoks 65 (full flowering: 50% of anthers matured) in spikes of wheat. The expression of *TraesCS6A02G020000* in spikes was higher (3.1 FPKM) at Zadoks 39 (flag leaf ligule visible). The highest expression in grain was observed for *TraesCS6A02G019800* (2.5 FPKM) at Zadoks 71 (kernel water ripe, no starch) and for *TraesCS6A02G020000* (4.1 FPKM) at Zadoks 85 (kernel soft dough) (Figure 4). Their expression patterns indicated that they could have biological roles in spike and grain, and more likely, spikelet development in wheat.

## Discussion

Yield gains associated with heterosis in wheat have been achieved at a slower pace than in other major crops such as maize and rice. The lack of an efficient system for producing hybrid seed is the major bottleneck impairing the competitiveness of hybrid breeding over line-breeding. At the breeding level, it hinders the efficient development of large numbers of test crosses required for the developing of heterotic pools. The CMS hybridization system based on sterility induced by the cytoplasm of *T. timopheevii* has been proven as a potentially efficient strategy for hybrid seed production. However, further genes and QTL controlling fertility restoration have to be identified and tagged with breeder-friendly molecular markers for utility in a more efficient breeding process. In wheat, this was severely constrained by its large complex genome of about 17 Gb, until the recent release of the reference sequence of the bread wheat variety *Chinese Spring* (IWGSC RefSeq v1.0, Appels et al., 2018). QTL for general fertility restoration in common wheat have been identified on chromosomes 1BS, 2AL, 2BS, 4BS, and 6AS (Ahmed et al., 2001), 1BS, 5A, and 7D (Zhou et al., 2005), 2DS (Dou et al., 2009), 1AS, 1BS, and 6BS (Geyer et al., 2018), and 2DS, 4BS, and 7AL (Yuan et al., 2020). In the present study, we performed QTL analyses in two populations segregating for restoration capacity contributed by 71R1203 and Gerek 79, for which a novel restorer locus on chromosome 6AS (*Rf9*) was identified, together with QTL on chromosomes 1AS, 1BS, 1DS, 2AL, 4AL, 5BL, and 6BS.

Gerek 79 was the most widely grown winter wheat cultivar in Turkey in the 1990s. It is a tall wheat derived from a Turkish landrace “Yala,” which has resistance to common bunt caused by *Tilletia caries* and *T. foetida* (Bonjean et al., 2001). 71R1203, developed from a cross of winter wheat “NB542437,” was one of the best restorer lines identified in field trials conducted in Eastern Washington during 1974–1978 (Allan and Rubenthaler, 1984). In BC_1_ populations derived from Gerek 79 and 71R1203, we found 12 QTL for seed set and number of sterile spikelets per spike. Four of these QTL (*QRf.lfl-6AS.1, QStS.lfl-6AS.1, QRf.lfl-6AS.2, QStS.lfl-6AS.2*) overlapped at the distal end of chromosome 6AS. Since the development of an immortal population which is segregating for restorer loci is hardly feasible in the cytoplasm of *T. timopheevii* (Geyer et al., 2018), QTL mapping in this study was performed using non-replicated trials, which limits the estimation of phenotypic variance components and testing of the QTL stability across diverse environments. This approach was also demonstrated efficiently and was successful for mapping QTL and major genes *Rf1* (Geyer et al., 2018) and *Rf8* (Sinha et al., 2013). Ahmed et al. (2001) detected a minor QTL for seed fertility on chromosome 6AS, but located much closer to the centromere using recombinant inbred lines derived from a cross of *T. aestivum* cv. ‘Chinese Spring’ and *T. spelta* var. *duhamelianum*.

It seems the new restorer locus *Rf9* that coincided with the QTL peaks of *QRf.lfl-6AS.1* and *QStS.lfl-6AS.1* in the subtelomeric region of chromosome 6AS (Figure 2A) is new as no *Rf* gene has been previously reported in the region. Putatively annotated high confidence genes located between the flanking markers of the gene *Rf9* showed possible associations with the candidate genes coding for mTERF proteins with higher expression patterns in the spikes and grains (Figure 4). Members of the mTERF family seem to be involved in fertility restoration in cereals. Pan et al. (2019) identified the candidate gene *Zea mays small kernel 3 (Zmsmk3)*, which contained two mTERF motifs and was required for the intron splicing of mitochondrial *nad4* and *nad1* genes and kernel development. Genome-wide association studies in a multiparental mapping population in hybrid barley detected two mTERF proteins linked to the restorer locus *Rfm3* on the short arm of chromosome 6H (Bernhard et al., 2019). A group of candidate genes belonging to the PPR family was also identified in the physical region associated with *QRf.lfl-6AS.*1. PPR genes have been cloned and well-characterized as essential components for fertility restoration in rice (Hu et al., 2012) and sorghum (Klein et al., 2005). Rizzolatti et al. (2017) identified *Rfm1* in barley as a major restorer gene that was described for the CMS system *msm1* derived from *Hordeum vulgare* ssp. *spontaneum*. The annotation of the nucleotide sequence for the *Rfm1* restorer allele showed that the locus carries tandemly repeated genes encoding for PPR proteins of the PLS-DYW subfamily. This group of PPR genes is known to be involved in RNA editing in plant organelles but has not been identified as restorer genes yet. Since the predicted restorer proteins are based on the reference sequence of Chinese Spring, which may not reflect gene content in tested restorer lines, functional characterization of the *Rf9* locus identified in BC_1_ Gerek 79 deserves additional investigations. Molecular cloning of *Rf* genes could improve our understanding of cytoplasmic nuclear interactions and provide molecular tools to facilitate the development of novel restorer lines. Furthermore, targeted screening of recombinant protein fragments (Edfors et al., 2019) could be used for dissecting the potential role of each candidate and their reciprocal interactions in mitochondrial transcript processing in future studies.

In BC_1_ 71R1203, a genome-wide QTL scan revealed two overlapping major QTL, *QRf.lfl-1AS* and *QStS.lfl-1AS*, within the interval between *IWB7436* (1.6 cM) and *IWB31602* (22.9 cM) on chromosome 1AS (Figure 2B). The identified QTL probably corresponds to *Rf1* in line R3, which was reported by Livers (1964), Yen et al. (1969), and Geyer et al. (2018).

Composite interval mapping also detected minor QTL *QRf.lfl-4AL, QStS.lfl-2AL, and QStS.lfl-6BS* in BC_1_ Gerek 79 and *QRf.lfl-1BS, QRf.lfl-5BL*, and *QStS.lfl-1DS* in BC_1_ 71R1203 (Table 2). The two QTL on chromosomes 1BS and 6BS may be identical to previously identified minor QTL (Ahmed et al., 2001; Zhou et al., 2005; Geyer et al., 2018). A KASP assay developed based on the SNP *IWB72107* was used to determine whether QTL *QRf.lfl-1BS* could be associated with *Rf3* located on chromosome 1BS (Geyer et al., 2018). No genetic polymorphism at this SNP locus was found between the parental lines when compared to the marker genotype of Primepi as a reference for the *Rf3* allele (data not shown). This indicates that *Rf3* was not present in BC_1_ Gerek 79 or BC_1_ 71R1203. A comparison of flanking SNP markers and genetic distances also showed that none of these minor QTL was linked to a region associated with a major *Rf* gene previously detected (Ma et al., 1995; Ahmed et al., 2001; Zhou et al., 2005; Stojałowski et al., 2013).

Both BC_1_ populations exhibited moderate restoration potential leading to incomplete fertility in most of the individuals. Nevertheless, genetic components of restorer parental lines could improve fertility restoration when combined with other restorer loci. Robertson and Curtis (1967) identified modifier genes located on chromosomes 1B, 2A, 3D, 6A, and 6B in Chinese Spring wheat that prevented full restoration, even in the presence of *Rf1* and *Rf2*. Geyer et al. (2018) reported a significant effect of modifier loci located on chromosomes 1BS and 6BL on the penetrance of *Rf1*, however, such interactions between major genes and modifier loci were proposed in several studies as essential for full fertility restoration (Ahmed et al., 2001; Zhou et al., 2005; Stojałowski et al., 2013; Würschum et al., 2017).

Besides the need to change the autogamous reproduction of wheat to an outcrossing system, the production of commercial hybrid seed will require a stable and efficient hybridization system comprising an easily pollinated female parent and a male parent with the ability to completely restore fertility under most field conditions. Selection for male characteristics such as plant height, anther size, pollen viability and longevity, general and specific combining ability with the female parent(s), and genetic diversity is important for exploring the best pollinators. However, the emphasis should be on the integration of genetic factors that control fertility restoration comprising QTL and *Rf* genes for developing males through repeated backcrossing. Selection of males segregating for fertility restoration loci in *T. timopheevii* cytoplasm could be performed by test crossing and evaluation of the plants, particularly under conditions that might inhibit the expression of fertility restoration such as heat and drought (Bonjean et al., 2001). To reach an acceptable level of hybrid fertility, a combination of two to three genes should be sufficient. Therefore, restorer lines with effective major QTL and genes such as *Rf1*, *Rf3*, and *Rf9* could be tested for maximum fertility restoration. Minor QTL for fertility restoration could be used for improving the female lines. Understanding the mode of action between the candidate genes and their activity in suppressing CMS-inducing open reading frames may help in the development of functional and predictive markers for tracking fertility restoration loci toward increasing the efficiency of the breeding processes. The results of our study could be used for developing new genetic resources of restorer lines and marker-assisted breeding in hybrid wheat. Also, it provides new insights into the genetic mechanisms controlling fertility restoration in wheat with the cytoplasm of *T. timopheevii* and lays the groundwork for gene characterization, cloning, and manipulation in future fundamental studies.

## Data Availability Statement

The original contributions presented in the study are included in the article/Supplementary Material, further inquiries can be directed to the corresponding author/s.

## Author Contributions

FS constructed the genetic maps, performed QTL mapping, and wrote the manuscript. FS and AB collected the phenotypic data and carried out the genetic analysis. MG and AB conducted the preliminary experiments. MG and VM supported the genetic analysis and designing of the project. LH conceived the study, supervised the project, and gained funding. All authors discussed the results, read, edited and approved the final manuscript for publication.

## Conflict of Interest

The authors declare that the research was conducted in the absence of any commercial or financial relationships that could be construed as a potential conflict of interest.

## Footnotes

1 http://wheatpedigree.net/sort/show/21747

2 https://triticeaetoolbox.org/wheat/

3 https://wheat.pw.usda.gov/ggpages/wgc/98/Intro.htm

4 https://urgi.versailles.inra.fr/blast_iwgsc/blast.php

5 http://plants.ensembl.org/Triticum_aestivum/Info/Index

6 https://plants.ensembl.org/biomart/martview/714673a041bd810256b7d4eb91aafc2d

7 https://wheat.pw.usda.gov/WheatExp/

8 http://urgi.versailles.inra.fr/files/RNASeqWheat/

9 https://shigen.nig.ac.jp/wheat/komugi/genes/symbolClassListAction.do?geneClassificationId=68

## Supplementary Material

Supplementary Table 1 Primer sequences designed for *KASP_IWB72107* marker associated with the gene *Rf3*.

Supplementary Table 2 Position of the markers (cM) in 21 linkage groups of wheat in BC_1_ Gerek 79 mapping population.

Supplementary Table 3 Position of the markers (cM) in 21 linkage groups of wheat in BC_1_ 71R1203 mapping population.

Supplementary Table 4 List of the potential candidate genes associated with QTL *QRf.lfl-6AS.1*, harboring *Rf9*, including flanking SNP markers, physical position, strand, gene ID, and functional description.
